# Probiotic Properties of Exopolysaccharide-Producing Bacteria from Natto

**DOI:** 10.1155/2023/3298723

**Published:** 2023-01-31

**Authors:** Vongsathorn Ngampuak, Acharawan Thongmee, Napapan Pongpoungphet, Kanda Wongwailikhit, Panan Kanchanaphum

**Affiliations:** ^1^Microbiology Unit, Department of Biomedical Science, Faculty of Science, Rangsit University, Patumthani, Thailand; ^2^RSU Scientific and Technology Research Equipment Center, Rangsit University, Patumthani, Thailand; ^3^Department of Chemistry, Faculty of Science, Rangsit University, Patumthani, Thailand; ^4^Biochemistry Unit, Department of Biomedical Science, Faculty of Science, Rangsit University, Patumthani, Thailand

## Abstract

Natto is a traditional Japanese food made from soybeans fermented with *Bacillus subtilis* var. *natto*. It is also a famous food in Thailand. Potential probiotics were screened from natto. *Bacillus subtilis* strain VN5 produced the most quantity of exopolysaccharide (EPS), so it was selected to study the properties of microbial EPS and probiotics. The Fourier transform infrared spectrometer or FT-IR spectroscopy confirmed the presence of carboxyl and hydroxyl groups. The patterns of FT-IR and levans are similar. The basic properties of probiotics were revealed. The 90% of VN5 strain resisted lysozyme within 30 min. VN5 survived under acidic conditions (pH 1-6), and the survival rate in 0.3%, 0.5%, and 1% bile solutions for 24 h was 100%. Unfortunately, VN5 did not inhibit the growth of *Escherichia coli*, *Staphylococcus aureus*, and *Salmonella typhi*. Gamma hemolysis was determined in VN5 strain. The finding on *Bacillus subtilis* strain (VN5) from natto paves the way to a high potential, useful new strain of probiotics.

## 1. Introduction

Microbial exopolysaccharides (EPS) are the outer cellular high atomic mass metabolites excreted by microorganism such as bacteria, yeasts, fungi, molds, and blue-green algae [1]. EPS plays an important role against many cellular functions, such as cell eating, phage defense, osmatic stress aggregation of bacterial cells, and surface adherence [2]. They could either be found as capsular polysaccharides which are covalently linked to cell surface or loosely bound or secreted outside during cell growth [3, 4]. EPS are used for several medical and industrial applications as medical coating devices, scaffolds, drug delivery systems, surgical sealants [5], gelling agents or biostabilizers [6], depollution agents, antioxidants, anti-inflammatory agents [7], antithrombotics, and anticancer agent [8]. In the food industry, they are used as stabilizers, flavorings, color carriers, and food thickeners [9]. In the last decade, several microorganisms have been proposed as potential EPS producers. Moreover, gram-positive *Bacillus* species have been potential as EPS-producing bacteria [10].

Probiotics are live microorganisms, which, when administered in adequate amounts, confer a health benefit on the host. Microbes used as probiotics are derived from different genera and species.

Natto is one of the most favorite fermented foods in Asia. They contain several kinds of useful probiotics which can stimulate the immune system and inhibit the growth of pathogen [11]. Moreover, some probiotics may secrete the EPS [12].

This study investigated potential bacteria in natto which could produce the EPS and evaluated probiotic properties of isolated bacteria and the characterization of EPS.

## 2. Material and Methods

### 2.1. EPS-Producing Bacteria Screening and Isolation

#### 2.1.1. Bacteria Screening

Fermented bean curd and natto were obtained from a market in Chiang Mai Province, Thailand. A 10-fold dilution of the fermented bean curd and natto was performed with distilled water. After that, they were incubated at 37°C for 20 min, spread on tryptic soy agar (TSA) (Becton Dickinson GmbH, Germany) plates, and incubated at 37°C for 18 h. The colonies were picked and used for a further study.

#### 2.1.2. Identification of Bacteria

Isolated pure colonies of bacterial culture were identified by 16S rRNA as described by Dorn-In et al. [13]. The DNA was extracted using a GF-1 Bacterial DNA Extraction kit (Vivantis). The polymerase chain reaction (PCR) was performed in a BIO-RAD MJ Mini Personal Thermal Cycler. The cycle conditions consisted of a single initial denaturation at 95°C for 5 min, followed by 40 cycles of 95°C for 30 sec, 55°C for 30 sec, and 72°C for 30 sec and final extension at 72°C for 5 min, respectively. The amplicon was sent to Solutions for Genetic Technologies in South Korea for sequencing.

First, the resulting sequences were checked and aligned using the BioEdit 7.0 sequence alignment editor (Isis Pharmaceuticals, Inc., Carlsbad, CA, USA). Then, they were compared with a homologous sequence stored on the GenBank database. Finally, the Basic Local Alignment Search Tool (BLAST) program, downloaded from the National Center for Biotechnology Information (NCBI) website, was used to evaluate the sequences. The MEGAX version 10.1.8 was used for constructing a phylogenic tree. Through the neighbor joining method by which a 1,000 bootstrap value was set, 16S rRNA of *Staphylococcus aureus* strain DSM 20231 (MN652637) was an outgroup gram-positive *Bacillus*, and *Escherichia coli* strain JCM1649 (AB24291) was an outgroup gram-negative *Bacillus*.

### 2.2. Bacterial Growth Condition and EPS Production

The isolated bacteria were cultured in tryptic soy broth (TSB) (Becton Dickinson GmbH, Germany) with 20% sucrose added. The pH value of the cultures was adjusted to 6.8 and allowed to grow at 37°C, for 24 hr. The growth curve was then measured. EPS production was carried out by using a 30 ml TSB medium with 20% sucrose in a 100 ml Erlenmeyer flask in batch culture. One ml of inoculum was added to the media. The inoculum had a cell count of about 10^6^ cells/ml^−1^. After that, it was incubated in a shaking incubator at 200 rpm for 48 hr at 37°C.

### 2.3. Isolation of EPS

After the cultivation of bacteria in the TSB, the cell culture was centrifuged at 5,000 g for 10 min. The supernatant was collected. Then, the 3 volumes of 95% ethanol were added, and the cell culture was stored at 4°C for 24 hr. After that, the solution was centrifuged at 12,000 g for 5 min at 4°C for precipitating the EPS polymer. Consequently, the polymer was washed with 70% ethanol, and the remained ethanol evaporated at room temperature.

### 2.4. Characterization of EPS by FT-IR Analysis of Crude-Purified EPS

The 10 mg EPS sample was homogenized and analyzed using Spectrum 100 Optica-PerkinElmer with a frequency range of 4,000-650 cm^−1^.

### 2.5. Basic Properties of Probiotic

#### 2.5.1. Lysozyme Tolerance Activity of the Isolated Bacteria

The bacterial cells were tested for lysozyme tolerance activity by using sterile electrolyte solution (SES; CaCl_2_ 0.22 g/l, NaCl 6.2 g/l, KCl 2.2 g/l, and NaHCO_3_ 1.2 g/l) and lysozyme 100 mg/l [14] and incubated at 37°C for 5, 15, 30, and 60 min, respectively. After that, cell counting was performed. The control group comprised bacterial cells cultured in nonlysozyme condition.

#### 2.5.2. Acidic Tolerance Activity of the Isolated Bacteria

The bacterial cells were tested for acidic tolerance activity by culturing the cells in nutrient broth or NBs (Becton Dickinson GmbH, Germany) of which pH values were adjusted to 1, 2, 3, 4, 5, and 6 by 1 N HCl. Then, they were incubated at 37°C for 24 hr, and counted cells (CFU/ml) were compared with those in the control group (culture in TSB pH 7) [15, 16].

#### 2.5.3. Bile Tolerance Activity of the Isolated Bacteria

The bacterial cells were tested for bile tolerance activity by culturing the cells in NBs which contained 0.3%, 0.5%, and 1% bile. Then, they were incubated at 37°C for 24 hr. After that, 100 *μ*l of the bacterial solution was used to count cells (CFU/ml) compared with the control group (cultured without bile) [17].

#### 2.5.4. Pathogenic Bacterial Inhibition of the Isolated Bacteria by the Well Diffusion Method

We used an agar well diffusion assay adapted from Barefoot and Klaenhammer [18]. The isolated bacteria were cultured in nutrient agar (NA) and incubated at 37°C for 24 hr. Subsequently, the pathogenic bacteria used in this study were *Escherichia coli*, *Staphylococcus aureus*, and *Salmonella typhi*. Each pathogenic bacterium was placed on an isolated bacteria plate using a 3-way swab technique, and the agar was punctured. The diameter of the punctured well was about 0.5 cm. Then, 50 *μ*l bacterial solution in each well was taken (the concentration of the cell count was about 10^6^ cells/ml^−1^), and the bacteria were cultured in the NB medium at 37°C for 24 hr. An inhibition zone was determined by observing a clear zone around the punctured well.

#### 2.5.5. Hemolysis Assay

The hemolysis testing was conducted on BD™ Columbia agar with 5% sheep blood (Becton Dickinson GmbH, Germany) to determine the type of hemolysis. VN2, VN3, VN5, and VN7 were cultured in BD™ Columbia agar with 5% sheep blood agar and incubated at 37°C for 24 hr for the determination of the hemolysis pattern. *Streptococcus pneumoniae*, *Streptococcus pyogenes*, and *Staphylococcus epidermidis* were used as the positive control of *α*-hemolysis, *β*-hemolysis, and *γ*-hemolysis, respectively.

## 3. Results

### 3.1. Isolation and Identification of Bacteria

**Figure 1:**
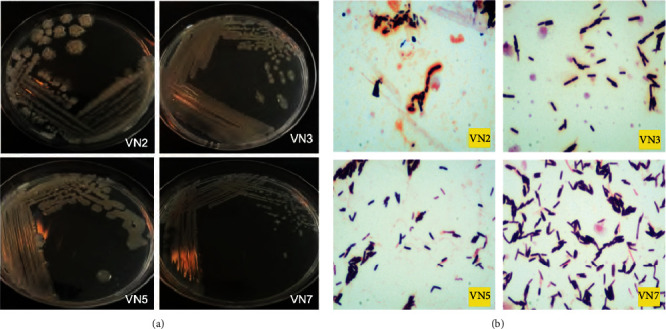
(a) Colony of VN2, VN3, VN5, and VN7. (b) Microscope image of rod shapes of VN2, VN3, VN5, and VN7.

**Figure 2:**
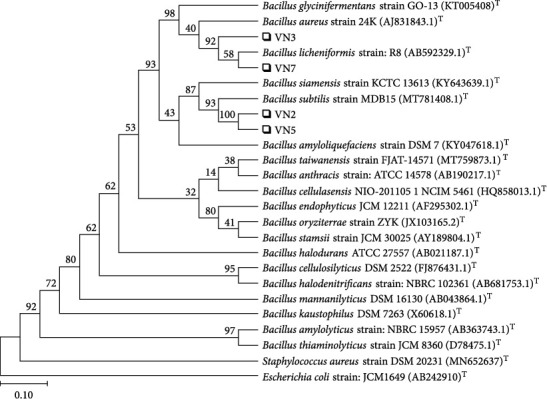
Phylograms of 16S rRNA sequence-based phylogenic trees of VN2, VN3, VN5, and VN7.

There were 4 isolated colonies, which were selected in the EPS production step as shown in Figure 1(a). VN2, VN3, VN5 and VN7 were white, slim and glossy. All of them had rod shapes as shown in Figure 1(b). After sequencing the 16S rRNA gene, the alignment of this gene is shown in Table 1 and Figure 2.

Table 1 and Figure 2 show that VN2 and VN5 were *Bacillus subtilis*, VN3 was *Bacillus aureus*, and VN7 was *Bacillus licheniformis.*

### 3.2. Bacterial Growth Condition and EPS Production

**Figure 3:**
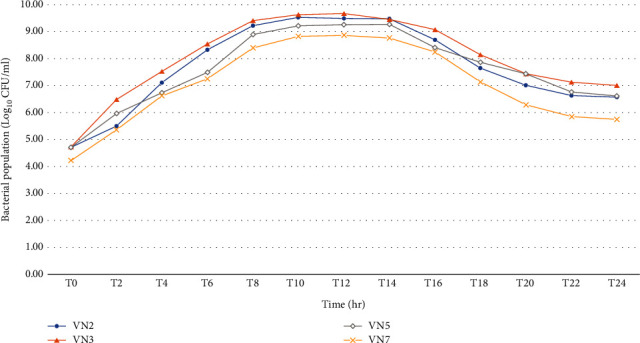
Growth curve of the isolated bacteria for 24 hr.

Figure 3 shows the exponential phases of VN2, VN3, VN5, and VN7. The exponential phases of VN2, VN3, VN5, and VN7 were 89.62 min, 100.96 min, 40.99 min, and 67.18 min, respectively. So it meant that VN5 grew most rapidly to the log phase while VN3 grew most slowly. However, the growth rate of VN3 was the best while VN7 was the worst.

**Figure 4:**
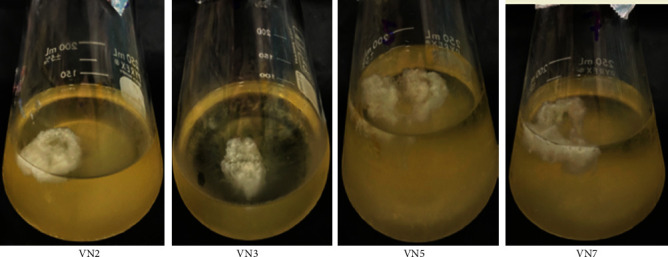
Polymers of EPS in the 30 ml cultures of VN2, VN3, VN5, and VN7, respectively.

After the EPS production from VN2, VN3, VN5, and VN7, EPS precipitation was performed as shown in Figure 4. The polymers of EPS in all cultures were observed, and the white fluffy particle appeared on the top of the cultures. The dry weights of EPS from VN2, VN3, VN5, and VN7 were 3.2, 1.07, 7.2, and 3.47 g/l, respectively.

### 3.3. FT-IR Spectral Analysis

**Figure 5:**
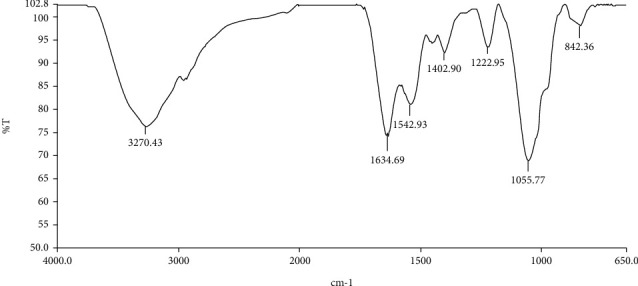
FT-IR analysis of VN5 EPS.

Fourier transform infrared spectroscopy (FT-IR) was used to identify the functional groups of the EPS produced from bacteria. FT-IR spectra were recorded from 4,000 cm^−1^ to 650 cm^−1^ to identify the functional groups of EPS from VN5 as shown in Figure 5. There were 7 major peaks that are shown in Figure 5.

### 3.4. Basic Properties of Probiotics

#### 3.4.1. Lysozyme Tolerance Activity

**Figure 6:**
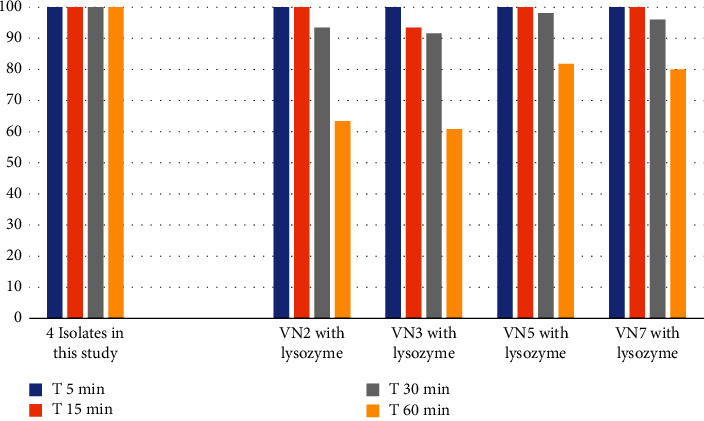
Lysozyme tolerance activity of VN2, VN3, VN5, and VN7, respectively.

From Figure 6, all VN2, VN3, VN5, and VN7 resisted lysozyme within 15 minutes. However, after 30 minutes of lysozyme incubation, the percentages of the surviving bacteria slightly dropped by 2-8%.

#### 3.4.2. Acidic Tolerance Activity

**Figure 7:**
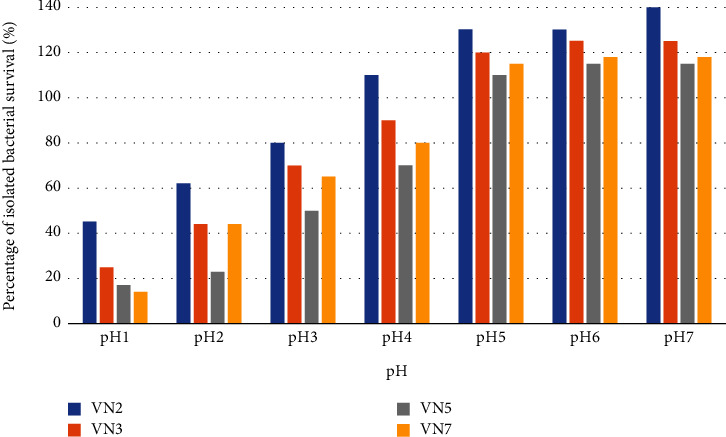
Acidic tolerance activity.

From Figure 7, VN2, VN3, VN5, and VN7 survived in all acidic environments (pH 1-6) after incubated for 24 hr. Especially, VN2 was the best survivor strain which could grow in lower pH values.

#### 3.4.3. Bile Tolerance Activity

**Figure 8:**
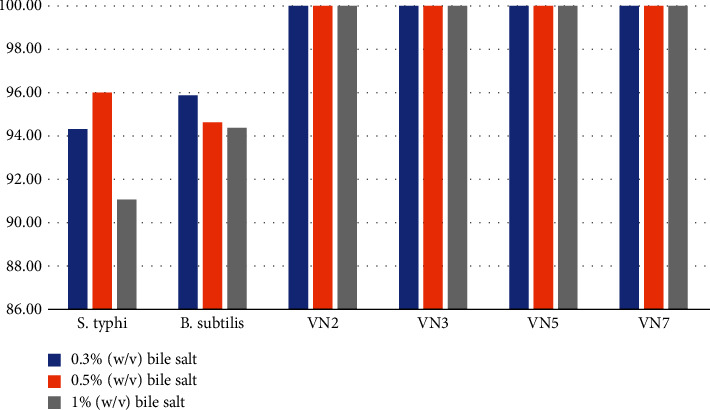
Bile tolerance activity.

All bile concentration did not affect the bacterial growth as shown in Figure 8. When compared with *Salmonella typhi* (representation of gram-negative bacteria) and *Bacillus subtilis* (representation of gram-positive bacteria), bile could retard the growth of both bacteria.

#### 3.4.4. Pathogen Bacterial Inhibition by Well Diffusion Method

**Figure 9:**
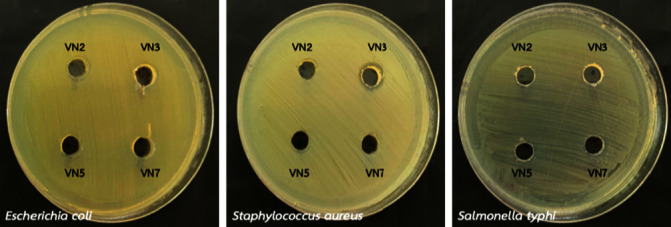
Pathogen bacterial inhibition of VN2, VN3, VN5, and VN7, respectively.

Figure 9 shows that no inhibition zones were observed since all cultures (VN2, VN3, VN5, and VN7) could not inhibit *Escherichia coli*, *Staphylococcus aureus*, and *Salmonella typhi*.

#### 3.4.5. Hemolysis Assay

**Figure 10:**
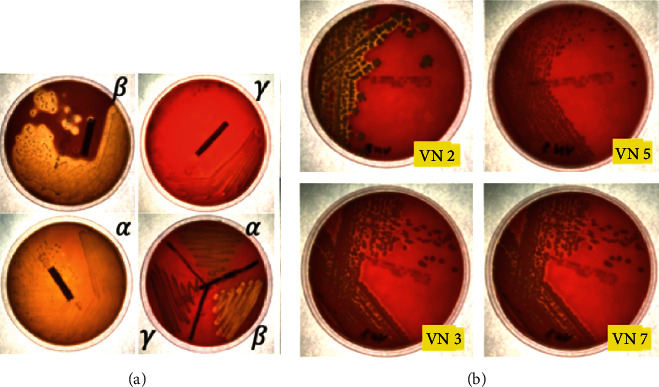
The control of three types of hemolysis (a). Hemolysis assays of VN2, VN3, VN5, and VN7, respectively (b).

From the blood agar, VN2 and VN3 strains were *α*-hemolysis as shown in Figure 10 while VN5 and VN7 were *γ*-hemolysis in which there was no change in the agar under and surrounding the colonies.

## 4. Discussion

In this study, the isolated bacteria from fermented bean curd and natto were *Bacillus subtilis* (VN2 and VN5), *Bacillus aureus* (VN3) and *Bacillus licheniformis* (VN7). Our results were similar to the study of Dos Santos et al. that [9] natto contained *Bacillus subtilis* [9, 19]. Noteworthy, Dimidi et al. [20] and Takagi et al. [21] studied probiotics in fermented soy and fermented bean curd and found that the fermented foods contained *Lactobacillus acidophilus*, *Lactobacillus bulgaricus*, *Streptococcus lactis*, *Bacillus subtilis*, and *Bacillus amyloliquefaciens*. Natto is a traditional Japanese fermented soybean produced through the fermentation of cooked soybeans with *Bacillus subtilis*. The fermentation of natto produces a number of bioactive factors such as nattokinase, bacillopeptidase F, vitamin K_2_, and dipicolinic acid [22]. Particularly, nattokinase is an enzyme of the subtilisin family produced by *Bacillus subtilis* [23] and can be isolated from natto [24]. Nattokinase has direct in vitro [24] and in vivo [25] fibrinolytic enzyme activity, increasing tissue plasminogen activators [26] and reducing platelet aggregation [27].

Noteworthy, the TSB with 20% sucrose added was the bacterial culture medium used to produce EPS in this study because Trabelsi et al. [28] reported that the sucrose was favorably used by many *Bacillus* species. Shih et al. [29] showed that *Bacillus subtilis* can produce ELS in the sucrose-rich growth medium. In addition, Lee et al. [30] reported that *Bacillus amyloliquefaciens* grew well in LB broth containing 0.3% oxgall. Another evidence that showed the importance of sucrose on media for EPS production in *Bacillus subtilis* is the study of Shih et al. [31]. They suggested that in the sucrose-rich environments, *Bacillus subtilis* (natto) Takahashi, a commercial natto starter, is able to selectively produce up to 50 g/l of EPS levan during batch fermentation [31]. The EPS found in this study may be levan for the reason that it was produced from *Bacillus* species, especially *Bacillus subtilis* [9]. In addition, the pattern of FT-IR of the EPS of VN5 was similar to levan [9, 32]. Levan is an EPS predominantly composed of D-fructose residues joined by glycosidic bonds *β* (2→1) and terminal glucose residue [9, 33]. The levan has wild industrial and technological applications. It highlights on the food industry as a stabilizer, flavor, color carrier, and food thickener [34]. After determining the function groups of EPS by FT-IR, there were 7 major peaks. A broad peak was observed at 3,270.43 cm^−1^ due to the presence of hydroxyl groups, and the polysaccharide characteristics of *Bacillus tequilensis*EPS were confirmed [35, 36]. The peak at 1,634.69 cm^−1^ revealed the presence of the carboxylate group and the characteristics of the IR absorption frequency band of polysaccharides [33]. The absorption peaks at 1,542.93 cm^−1^ and 1,402.90 cm^−1^ were the symmetric stretching vibration of carboxyl groups. The bands at 1,22.95 cm^−1^ and 1,055.77 cm^−1^ were attributed to the stretch of C-O and C-O-C which ascertained the existence of polymer [36, 37]. The peak at 842.36 cm^−1^ represented the characteristic peak of heteropolysaccharide compounds [37].

To assess probiotic potential, VN2, VN3, VN5, and VN7 were tested in acidic environment, bile and lysozyme tolerance, and pathogen inhibition activity. All VN strains could survive in acidic environment. The finding was consistent with the finding of Lee et al. [30] that *Bacillus amyloliquefaciens* LN survived in the culture media at pH 2.0 for 3 hr. In addition, another probiotic, *Leuconostoc mesenteroides*, had the survival rates more than 80% in acidic environments (pH 2 and 3) [38]. In bile tolerance activity, VN 2, 3, 5, and 7 demonstrated absolute bile tolerance in 0.3, 0.5, and 1% bile which was noticeable. This result was similar to Walker and Gilliland [39] and Vinderola and Reinheimer [40]. The bacteria which have high tolerance to bile salt were effective in bile salt deconjugation and consequently effective in lowering serum cholesterol. Another interesting property of probiotic is lysozyme tolerance. Özkan et al. and Mengesha et al. [41, 42] revealed that *Lactobacillus* (L.) strains isolated from traditional Turkish Tulum cheeses showed moderate lysozyme tolerance while VN2, VN3, VN5, and VN7 demonstrated strong lysozyme tolerance in 30 min and moderate tolerance in 60 min similar to Özkan et al. [41]. Furthermore, Guariglia-Oropeza and Helmann [43] mentioned that the extracytoplasmic factor on (ECF) sigma (*σ*) factors especially *σ*^*w*^ had involved in lysozyme resistance property. The *σ*^*w*^ regulon includes at least 60 genes that inactivate, sequester, or eliminate toxic compounds form cell, and its expression is induced by variety of cell envelope active compounds, detergent, and alkali stress ([44–46]). It meant that the mechanism for lysozyme resistance in Bacillus is very complicated. Unfortunately, VN2, VN3, VN5, and VN7 did not show antibacterial activity in *Escherichia coli*, *Staphylococcus aureus*, and *Salmonella typhi*, while *Bacillus subtilis* isolated from natto by Dimidi et al. [20] revealed that this bacteria was the important factor in treating *Streptococcus pneumoniae* infections. Therefore, in further studies, *Streptococcus pneumoniae* could be performed for antibacterial activity testing. For safety assessment, all VN strains showed gamma hemolysis identifying that they were safe.

## 5. Conclusion

In this study, *Bacillus subtilis* strain (VN5) isolated from natto in the northern part of Thailand may be a good candidate for probiotic bacteria because it could produce a high amount of EPS which has the same FT-IR pattern of levan. In addition, this VN5 strain had basic properties of probiotic such as lysozyme tolerance, acidic tolerance, bile tolerance, and safety. So, VN5 and its EPS will allow the feasibility of the industrial applications in the future.

## Figures and Tables

**Table 1:** The accession number of VN2, VN3, VN5, and VN7.

Colony number	Submission	Isolates	Accession number
VN2	SUB9848071	*Bacillus subtilis* strain VN2 16S rRNA gene, partial sequence	MZ389241
VN3	SUB9849022	*Bacillus aureus* strain VN3 16S rRNA gene, partial sequence	MZ389932
VN5	SUB9849390	*Bacillus subtilis* strain VN516S rRNA gene, partial sequence	MZ389888
VN7	SYB9849510	*Bacillus licheniformis* strain VN7 16S rRNA gene, partial sequence	MZ389979

## Data Availability

All data used to support the findings of this study are included within the article.
